# Anterior temporal lobectomy and selective AmygdaloHippocampectomy complications across Europe: review, meta-analysis, and Delphi consensus

**DOI:** 10.1016/j.bas.2025.104304

**Published:** 2025-06-18

**Authors:** Maria D. Karagianni, Olaf E.M.G. Schijns, Alexandros Brotis, Anastasia Tasiou, Christian Auer, Thomas Blauwblomme, Marcelo Budke, Alexandre Rainha Campos, Santiago Candela-Cantó, Hans Clusmann, Alessandro Consales, Massimo Cossu, Daniel Delev, Christian Dorfer, Georg Dorfmüller, Arild Egge, Lorand Eröss, Sarah Ferrand-Sorbets, Flavio Giordano, Cihan Isler, Jugoslav Ivanovic, Thilo Kalbhenn, Atte Karppinen, Paivi Koroknay-Pal, Niklaus Krayenbühl, Marec von Lehe, Carlo E. Marras, Ioannis Mavridis, Daniel Nilsson, Julia Onken, Christian Raftopoulos, Michele Rizzi, Dirk van Roost, Karl Rössler, Jonathan Roth, Jordi Rumia, Alexandra Seromenho-Santos, Thomas Sauvigny, Didier Scavarda, Christian Scheiwe, Karl Schaller, Sophie Schuind, Ido Strauss, Tom Theys, Mustafa Uzan, Konstantinos N. Fountas, the EANS Functional Neurosurgery section, Franziska Schmidt, Linda Ackermans, Pedro Duarte Batista, Michael Hart, Marie Krüger

**Affiliations:** ao Department of Neurosurgery, University of British Columbia, Vancouver, Canada; ap Department of Neurosurgery, Maastricht University Medical Center, the Netherlands; aq Department of Neurosurgery, Centro Hospitalar Universitário de Lisboa Norte, Lisboa, Portugal; ar Department of Neurosurgery, New Victoria Hospital, London, UK; as Department of Neurosurgery, National Hospital for Neurology and Neurosurgery, London, UK; a Department of Neurosurgery, University General Hospital of Larissa, Biopolis, Larissa, 41110, Greece; b Department of Neurosurgery, Maastricht University Medical Center, Maastricht, the Netherlands; c Academic Center for Epileptology, Maastricht University Medical Center and Kempenhaeghe, Maastricht-Heeze, the Netherlands; d Mental Health and Neuroscience (MHeNs) Research Institute, University Maastricht (UM), Maastricht, the Netherlands; e Department of Neurosurgery, Johannes Kepler University Linz, Kepler University Hospital, Linz, Austria; f Department of Pediatric Neurosurgery, Hôpital Necker, Université de Paris, Paris, France; g Department of Neurosurgery, Hospital Infantil Universitario Nino Jesus, Madrid, Spain; h Department of Neurosurgery, Hospital Santa Maria, Lisbon, Portugal; i Department of Neurosurgery, Hospital Sant Joan de Déu Barcelona, Barcelona, Spain; j Department of Neurosurgery, RWTH Aachen University Hospital, Aachen, Germany; k Department of Pediatric Neurosurgery, Istituto G. Gaslini, Genoa, Italy; l "Claudio Munari" Centre for Epilepsy Surgery, Department of Neuroscience, ASST Grande Ospedale Metropolitano Niguarda, Milan, Italy; m Department of Neurosurgery, Medical University of Vienna, Vienna, Austria; n Department of Neurosurgery, Hopital Fondation Adolphe de Rothschild, Paris, France; o Department of Neurosurgery, Oslo University Hospital, Oslo, Norway; p Department of Neurosurgery, National Institute of Mental Health, Neurology and Neurosurgery, Budapest, Hungary; q Department of Neurosurgery, Meyer Children's Hospital IRCCS, University of Florence, Italy; r Department of Neurosurgery, Istanbul University-Cerrahpasa, Istanbul, Turkey; s Department of Neurosurgery, Bielefeld University, Medical School, Bielefeld, Germany; t Department of Neurosurgery, University of Helsinki, Helsinki University Hospital, Helsinki, Finland; u Department of Neurosurgery, Universitäts-Kinderspital Zürich-Eleonorenstiftung, Zürich, Switzerland; v Department of Neurosurgery, Ruppiner Kliniken, Neuruppin, Germany; w Department of Neurosurgery, Ospedale Pediatrico Bambino Gesu, Roma, Italy; x Department of Neurosurgery, University General Hospital of Alexandroupolis, Alexandroupolis, Greece; y Department of Neurosurgery, Sahlgrenska University Hospital, Göteborg, Sweden; z Department of Neurosurgery, Charite University Hospital, Berlin, Germany; aa Department of Neurosurgery, Clinique Universitaires Saint Luc, Brussels, Belgium; ab Functional Neurosurgery Unit and Epilepsy Surgery Program, Department of Neurosurgery, Fondazione IRCCS Istituto Neurologico "Carlo Besta", Milan, Italy; ac Department of Neurosurgery, University Hospital Ghent, Ghent, Belgium; ad Department of Neurosurgery, Tel Aviv Sourasky Medical Center, Tel Aviv, Israel; ae Department of Neurosurgery, Centro Hospitalar de Lisboa Ocidental, Lisbon, Portugal; af Department of Neurosurgery, University Medical Center Hamburg-Eppendorf, Hamburg, Germany; ag Department of Neurosurgery, Hopital La Timone Enfants, Marseille, France; ah Department of Neurosurgery, University Hospital, Freiburg, Germany; ai Department of Neurosurgery, Geneva University Medical Center, Geneva, Switzerland; aj Department of Neurosurgery, Hopital Erasme, Brussels, Belgium; ak Functional Neurosurgery Unit, Tel Aviv Sourasky Medical Center, Tel Aviv, Israel; al Department of Neurology and Neurosurgery, School of Medicine, Tel Aviv University, Tel Aviv, Israel; am Department of Neurosurgery, University Hospital Leuven, Leuven, Belgium; an Faculty of Medicine, School of Health Sciences, University of Thessaly, Biopolis, Larissa, 41110, Greece

**Keywords:** Complications, Epilepsy surgery, Anterior temporal lobectomy, Selective amygdalohippocampectomy, Morbidity, Mortality

## Abstract

**Introduction:**

Epilepsy is a neurological disorder affecting over 50 million people globally, with around 30 % of them classified as having drug-resistant epilepsy (DRE). Temporal lobe epilepsy (TLE) is the most frequently encountered type of surgically treated epilepsy. The primary surgical approaches for TLE include anterior temporal lobectomy (ATL) and selective amygdalohippocampectomy (selAH).

**Research question:**

This study sought to gather expert European consensus on surgical strategies and complication rate for ATL and selAH, in both adult and pediatric patients.

**Materials and methods:**

A modified Delphi technique was employed, with 39 experienced epilepsy surgeons from 35 different European centers. A 22-item questionnaire addressed key surgical considerations, including mortality, morbidity, neurological deficits, infection rates, and potential psychiatric and cognitive complications.

**Results:**

The survey had a 43 % response rate. Mortality rates for both surgical approaches ranged between 0 and 1 %. Visual field deficits (VFDs) were more frequently observed after ATL (over 16 %) compared to selAH (2–10 %). Permanent motor deficits were rare (<2 %), while complications such as infections and hematomas were reported in 0–2 % and less than 5 % of cases, respectively for both procedures. While psychiatric and cognitive complications were acknowledged, no consensus was reached regarding their prevalence or screening methods.

**Discussion:**

The results underscore the value of advanced imaging, thorough preoperative evaluation, and intraoperative monitoring. Future research is needed to refine outcome optimization and standardize training protocols.

**Conclusions:**

Consensus was achieved on critical aspects of surgical planning and complication management, providing support for the development of standardized practices in temporal lobe epilepsy surgery.

## Highlights

• Expert consensus on complications associated with ATL and selAH.
• ATL is associated more frequently with visual field deficits than selAH.
• There is strong consensus on the need for comprehensive neuropsychological testing pre- and post-operatively; however, variability remains in the tools and practices used.
• Proper imaging and intraoperative monitoring are crucial for better outcome.
• Standardized protocols and ongoing research are needed for better outcomes.

## 1 Introduction

Epilepsy, a chronic disease of the central nervous system, characterized by recurrent seizures, affects minimally 50 million individuals worldwide (approximately 1 % of the world population) (Zhang et al., 2023; Mehndiratta and Wadhai, 2015). It has been estimated that 30 % of the affected patients are being classified as having drug-resistant epilepsy (DRE) (Kwan et al., 2009; Dalic and Cook, 2016; Englot and Chang, 2014). It is also well known that temporal lobe epilepsy (TLE) is the most frequent type of focal epilepsy referred for surgery (Téllez-Zenteno et al., 2011bib_th_2011). Anterior temporal lobectomy (ATL) and selective amygdalohippocampectomy (selAH) are the two most established surgical interventions for managing DRE, particularly for adult patients with focal epilepsy originating in the temporal lobe. ATL involves the removal of the anterior segment of the temporal lobe, often including portions of the medial temporal structures i.e. hippocampus, amygdala, uncus, and the temporal part of the piriform cortex, aiming at eliminating the epileptogenic zone (EZ). Alternatively, selAH is a more targeted approach towards the mesial temporal structures, preserving much of the neocortical parts of the temporal lobe, with the aim to minimize cognitive and neurological side effects (Jud et al., 2024).

Numerous studies (Wiebe et al., 2001; Engel, 2012) have demonstrated that surgery for TLE provides substantial seizure control, with seizure freedom rates reported from 33 % to 93 % (median≈70 %) of patients with mesial temporal lobe epilepsy (mTLE) (Englot and Chang, 2014; McIntosh et al., 2001; Englot et al., 2013a, 2013b; Bjellvi et al., 2014). However, despite these favorable outcomes, both ATL and selAH carry different/various risks, including cognitive and psychiatric side effects, visual field deficits, and other neurological complications (Brotis et al., 2019). The rate of postoperative mortality remains low at approximately 0–1 %, though morbidity rates are notably higher, affecting up to 17 % of patients (Brotis et al., 2019). Characteristically, in the Swedish registry it is described that 2.9 % of the patients undergoing temporal lobectomy experienced major, while 7.8 % minor complications [13,14].

Recent refinements and advances in pre-operative multimodal imaging, intraoperative electrophysiology, and surgical technique have contributed to improved patient outcomes by minimizing complications (Eryurt et al., 2015; Winston et al., 2011). These include pre-operative advanced multimodal imaging [such as Diffusion Tensor Imaging (DTI)], expansion and enrichment of the human brain connectome, better cortical visualization due to stronger magnetic field (7T MRIs), and segmentation techniques, further development of task-generated and resting-state functional MRI. The anesthesiologic techniques have been progressively improved due to the expanding usage of awake mapping strategy and the improvement of the available pharmacological agents. Moreover, the routine employment of intraoperative monitoring (e.g., MEPs, SSEPs, cortical and subcortical stimulation and mapping), further accuracy improvement of neuronavigation, and refinement of surgical techniques mainly due to better visualization using exoscope, or microscope advanced magnification techniques, like for example augmented reality. These medical advances, generated by technological evolution, continued research, and refinement of surgical protocols lead to a higher quality of life in patients with DRE.

## 2 Objective

The primary aim of this initiative is to achieve a comprehensive consensus to provide colleagues performing epilepsy surgery with guidance for optimal surgical planning and a complication avoidance protocol, for ATL and selAH procedures in both adult and pediatric patients. The target audience for this consensus statement is neurosurgeons performing surgery for TLE.

## 3 Methodology

### 3.1 Modified Delphi method

A modified Delphi method, an iterative process involving the collection and distillation of anonymous expert opinions through a series of questionnaires, was employed to achieve a consensus (Nasa et al., 2021). This multi-stage approach included:

Round 1: At least one round of developing a focused questionnaire on the complications of ATL and its modifications, by a panel of responders, based on an extensive review of the pertinent literature. The questionnaire was built on Google Forms platform.
Round 2: The questionnaire was distributed by email to epilepsy surgeons practicing across Europe in 37 epilepsy surgery centers (Table 1), and to the panel-members of the European Association of Neurosurgical Societies (EANS) Functional Neurosurgery Section. In order to maximize recruitment, three reminder-emails were sent to participants.

**Table 1:** Table of responders.

Medical Center	Country
Department of Neurosurgery, Medical University of Vienna, Vienna	Austria
Department of Neurosurgery, Johannes Kepler University Linz, Kepler University Hospital, Linz ·	
Department of Neurosurgery, University Hospital Ghent, Ghent	Belgium
Department of Neurosurgery, University Hospital Leuven, Leuven	
Department of Neurosurgery, Clinique Universitaires Saint Luc, Brussels	
Department of Neurosurgery, Hopital Erasme, Brussels	
Department of Neurosurgery, Helsinki University Hospital	Finland
Department of Pediatric Neurosurgery, Hôpital Necker, Université de Paris, Paris	France
Department of Neurosurgery, Hopital Fondation Adolphe de Rothschild, Paris	
Pediatric Neurosurgery chez Hôpital d'Enfants de la Timone Marseille	France
Department of Neurosurgery, Medizinische Hochschule Brandenburg ·	Germany
Department of Neurosurgery, RWTH Aachen University Hospital, Aachen	Germany
Department of Neurosurgery, Charite University hospital, Berlin	
Department of Neurosurgery, University hospital, Freiburg	
Department of Neurosurgery, Bielefeld University, Medical School, Bielefeld, Germany	
Department of Neurosurgery, University Medical Center Hamburg-Eppendorf, Hamburg	
Department of Neurosurgery, University General Hospital of Larissa, Faculty of Medicine, School of Health Sciences, University of Thessaly	Greece
Department of Neurosurgery, University General Hospital of Alexandroupolis, Alexandroupolis	
Department of Neurosurgery, National Institute of Mental Health, Neurology and Neurosurgery, Budapest	Hungary
Department of Neurosurgery, Tel Aviv Sourasky medical center, Tel Aviv	Israel
Department of Neurosurgery, Meyer Children's Hospital IRCCS, University of Florence	Italy
Department of Neurosurgery, Ospedale Pediatrico Bambino Gesu, Roma	
Neurosurgery Unit, Giannina Gaslini Pediatric Hospital IRCCS, Genoa	
Fondazione IRCCS Istituto Neurologico “Carlo Besta”	
“C. Munari”, Niguarda Ca 'Granda Hospital., University of Genoa, Italy	
Department of Neurosurgery, Maastricht University Medical Center, Maastricht	The Netherlands
Department of Neurosurgery, Oslo University Hospital	Norway
Department of Neurosurgery, Hospital Santa Maria, Lisbon	Portugal
Department of Neurosurgery, Centro Hospitalar de Lisboa Ocidental, Lisbon	Portugal
Department of Neurosurgery, Hospital Infantil Universitario Niño Jesús, Madrid	Spain
Department of Neurosurgery, Hospital Sant Joan de Deu, Barcelona	
Department of Neurosurgery, Sahlgrenska University hospital, Göteborg	Sweden
Department of Neurosurgery, Universitäts-Kinderspital Zürich-Eleonorenstiftung, Zürich	Switzerland
Department of Neurosurgery, Geneva University Medical Center, Geneva	
Department of Neurosurgery, Istanbul University-Cerrahpasa, Istanbul	Turkey
Round 3: The consensus statements were generated based on the anonymous responses, with items achieving 70 % or greater agreement among the panelists considered to have reached a consensus.
Round 4: The consensus statements were subsequently reviewed and refined through additional rounds as needed to achieve a high level of agreement. The final consensus statements were synthesized into a summary document.
Round 5: Based on the feedback from the readers, future revisions and updates may be undertaken.

### 3.2 The panel of responders

The expert panel consisted of neurosurgeons from leading academic institutions and specialized epilepsy surgery centers across Europe, all with extensive experience in the management of DRE. The expert panel provided their collective expertise and guidance on the optimal surgical planning and complication avoidance strategies for ATL and selAH procedures, in both adult and pediatric populations.

### 3.3 Areas of interest

The expert panel was asked to provide a consensus on the following key domains:

1. Mortality and morbidity associated with ATL and selAH
2. Techniques and approaches to screen for and minimize surgical complications
3. Identification of potential risk factors predisposing to observed complications
4. Implementation of pre-, intra-, and post-operative protocols to improve patient outcomes

### 3.4 The questionnaire

Our questionnaire consisted of 22 items related to the aforementioned areas of interest. There were three open-ended questions, 10 Likert-scale items, and nine multiple-choice questions. One question was related to the mortality rate associated with ATL and selAH, five questions covered the observed frequency of complications associated with ATL and selAH, including visual field deficits (VFD), permanent motor deficits, the occurrence of postoperative infections and hematomas, as well as cognitive, language, and psychiatric postoperative manifestations. The remaining items focused on surgical planning, surgical technique, and intra- and post-operative management strategies to mitigate these complications.

### 3.5 Survey instrument

The questionnaire was designed to collect expert opinions from surgeons on complications related to **Anterior Temporal Lobectomy (ATL)** and **Selective Amygdalohippocampectomy (selAH)** in **children and adults**.

The 22-question instrument used a mix of multiple-choice (including predefined percentage ranges), Likert-scale, and open-ended formats. It systematically covered:

**Observed complication rates:** Mortality, visual, motor, psychiatric, cognitive, and speech/language deficits, as well as infection and hematoma rates, all stratified by patient age and surgical approach.

**Preventative and diagnostic strategies:** Measures to avoid specific deficits (visual, motor, hematoma) and the routine use of Transcranial Doppler (TCD) for vasospasm detection.

**Infection control practices:** Hair shaving, antibiotic prophylaxis strategies, and the perceived impact of surgical duration on infection.

**Pre/postoperative evaluations:** Use of psychiatric and neuropsychological assessments.

**Surgical experience:** Minimally required case numbers for different ATL/selAH procedures.

Divergent opinions were addressed through careful analysis and by engaging experts in structured discussions and clarifications to facilitate resolution.

The full questionnaire is available in the appendix (Supplementary material -Table 1).

## 4 Results

The overall response rate was 43 %. The questionnaire was distributed among epilepsy surgeons from 37 different centers. More specifically, 39 epilepsy surgeons responded.

### 4.1 Mortality – Q1

The survey received responses from 39 panelists on the mortality of ATL in children, 28 on selAH in children, 36 on ATL in adults, and 30 on selAH in adults. All of our responders reported that the mortality rate associated with ATL or selAH ranged in their series between 0 and 1 %, irrespectively of the patient's age and the implemented surgical technique.

### 4.2 Visual fields deficits - Q 2-4

The survey received responses from 38 panelists on visual field deficits associated with ATL in children, 28 on selAH in children, 35 on ATL in adults, and 29 on selAH in adults. While there was no consensus on the specific observed frequency of VFD, most of our panelists agreed that the complication rate was higher than 16 % in patients undergoing ATL. In contrast, selAH was thought to have a lower frequency of VFD, with an estimated incidence below 2 % in children, while the respective incidence was 5–10 % in adults. Likewise, a variety of maneuvers were recommended to reduce the frequency of VFDs, including careful surgical planning (intraoperative use of probabilistic tractography - DTI of Meyer's loop), intraoperative monitoring (such as Visual Evoked Potentials – VEPs), and even the consideration of less invasive surgical alternatives, such as limited neocortical resection. There was a consensus on the need to examine visual fields pre- and post-operatively, in both the early postoperative period and during a long-term follow-up (37/39). However, there was no consensus on the preferred examination method, with 25 out of 39 panelists recommending visual field perimetry, while 12 out of 39 recommending only clinical examination.

### 4.3 Motor deficits - Q 5 -7

The survey received responses from 38 panelists on motor deficits associated with ATL in children, 28 on selAH in children, 35 on ATL in adults, and 25 on selAH in adults. The majority of the panelists (60 %) agreed that permanent motor deficits following ATL are uncommon, occurring in 0–2 % of patients, whereas selAH was associated with an incidence of less than 2 %, in both adult and pediatric populations. With a few exceptions (16 %), the panelists emphasized the importance of utilizing advanced and precautionary measures to minimize the risk of permanent motor deficits, including the obtaining of a pre-operative functional magnetic resonance imaging (fMRI), the use of neuronavigation, intraoperative neurophysiological monitoring with somatosensory evoked potentials (SSEPs) and motor evoked potentials (MEPs), the application of direct cortical and subcortical stimulation, and meticulous surgical techniques with minimal use of bipolar coagulation. The agreement level reached as much as 84 %. Moreover, a consensus was reached on the role of intraoperative transcranial Doppler for predicting postoperative vasospasm. The vast majority (38/39) of our panelists advocated against its usage.

### 4.4 Infections - Q 8-12

The survey received 38 responses for ATL in children, 28 for selAH in children, 35 for ATL in adults, and 25 for selAH in adults. Our panelists reached a consensus and agreed that early and late post-operative infections occurred in approximately 2 % of cases, irrespectively of the employed surgical technique. However, it was suggested that infection rates could be even further reduced by using prophylactic antibiotics, particularly second-generation cephalosporins. Notably, 23.7 % of panelists opted to avoid shaving the patient's hair for surgery. Interestingly, approximately half of the panelists (51 %) believed that the duration of the surgery had no impact on the occurrence of postoperative infections.

### 4.5 Postoperative hematomas - Q 13-14

Regarding the incidence of postoperative hematomas, 38 panelists reported on ATL in children, 28 on selAH in children, 35 on ATL in adults, and 25 on selAH in adults. Their responses indicated a low rate of this complication, occurring in less than 5 % of cases, irrespectively of the performed surgical procedure. Our panelists emphasized the importance of meticulous surgical techniques to achieve adequate hemostasis, a strategy that obviates the need for routine wound drain placement.

### 4.6 Psychiatric changes - Q 15-17

The surveyed panelists, 38 for ATL in children, 28 for selAH in children, 35 for ATL in adults, and 25 for selAH in adults, reached a consensus that psychiatric changes following ATL and selAH occur at relatively low rates, less than 10 %, in children. However, there was no consensus on the occurrence of post-operative psychiatric manifestations in adults. The majority of the responders (53.8 %) would not recommend routine pre- and post-operative psychiatric evaluation, though 28 % felt it should be performed on an individual basis. Several batteries of psychiatric tests were recommended, including the Beck Depression Inventory, and the Beck Anxiety Inventory, but none was unanimously accepted.

### 4.7 Cognitive deficits - Q 18 -20

The surveyed panelists, 38 for ATL in children, 28 for selAH in children, 35 for ATL in adults, and 25 for selAH in adults, did not reach a consensus on the observed frequency of cognitive deficits after ATL in any age group. However, most of our panelists felt that the frequency of such deficits is lower after selAH. Panelists agreed (78.9 %) on the need for comprehensive neuropsychological testing to assess multiple cognitive domains pre- and post-operatively for developing proper individualized rehabilitation plans. Still, no single assessment tool achieved broad acceptance among our panelists.

### 4.8 Language deficits- Q 21

According to the surveyed panelists, encompassing 38 respondents for ATL in children, 28 for selAH in children, 35 for ATL in adults, and 25 for selAH in adults, language deficits were considered uncommon for either surgical approach in children. The majority (>72 %) of the participants reported an incidence of less than 2 %. However, there was a less robust agreement on the incidence of language deficits in adults, with a tendency for higher rates, reaching as high as 5 % for either ATL or selAH.

### 4.9 Achievement of surgeons’ competence - Q 22

According to the surveyed panelists, encompassing 38 respondents for ATL in children, 28 for selAH in children, 35 for ATL in adults, and 25 for selAH in adults, there was no agreement on the minimally required number of operations for achieving competence in ATL or selAH surgery. While some panelists emphasized that this depends on the individual surgeon's background and experience, others suggested a minimum number of required cases, ranging between 25 and 30 per year. Moreover, the prerequisite number of selAH procedures for achieving competency seemed to increase, due to the enhanced technical complexity of this procedure.

**Fig. 1:**
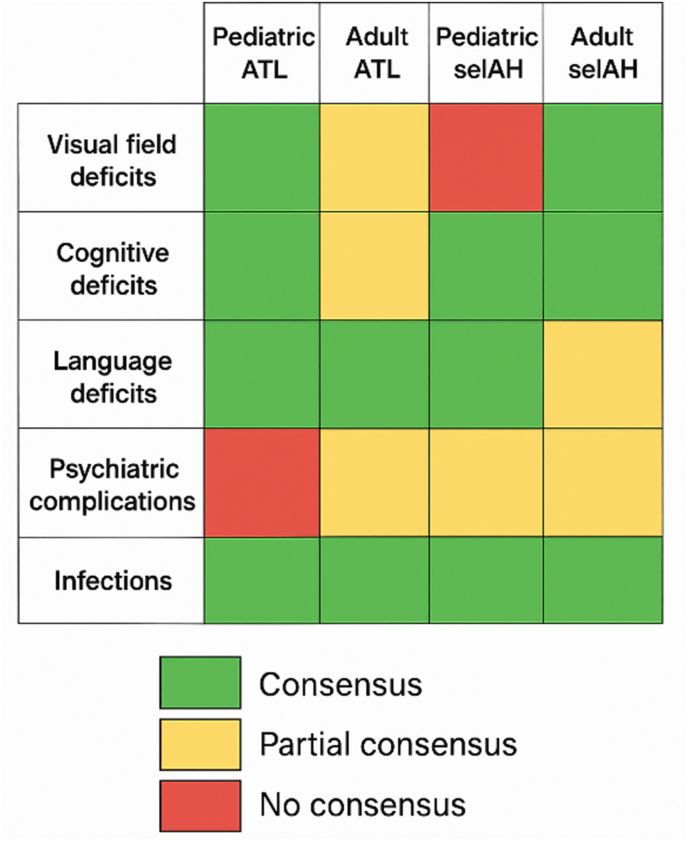
Overview of our findings.

All 22 questionnaire items, the item-wise consensus status across all rounds, as well as the detailed response frequencies and percentages are listed in the appendix (Supplementary Table 2). The key consensus outcomes are presented in Fig. 1 to provide a clearer overview of our findings.

## 5 Discussion

Anterior temporal lobectomy (ATL) and selective amygdalohippocampectomy (selAH) are the most commonly used surgical procedures to treat DRE. While these surgical methods offer promising results in controlling seizures, they are also associated with a broad range of potential complications. According to the current literature, postoperative mortality risk can be as high as 1 %, while cumulative morbidity rates may reach up to 17 % (Brotis et al., 2019). The entire spectrum of observed complications is usually classified in two large categories: neurological and non-neurological. The latter group usually includes craniotomy or resection-associated hematomas, superficial wound infection, bone-flap osteomyelitis, or central nervous system infections. Even though half of the panelists believe that the duration of the surgery does not have an impact on post-operative infections, a recent study (Liu et al., 2024) postulates that surgery duration is positively correlated with post-operative infection rate, especially if the procedure lasts longer than 4 h. The group of neurological complications consists of hemiparesis, language deficits, cranial nerve injuries, visual field deficits, cognitive disorders, psychiatric or behavioral disturbances, hydrocephalus, and post-resection cyst formation (Brotis et al., 2019; Popovic et al., 1995; Sindou et al., 2005; Georgiadis et al., 2013). Severe complications such as hemiparesis and language disorders can occur in as high as 4 % of patients undergoing ATL or selAH (Brotis et al., 2019; Mathon et al., 2017). It has to be mentioned, however, that this percentage may be higher if a thorough, neurocognitive evaluation is post-operatively performed, particularly when the intervention is on the dominant side. In a study published by Busch et al. (Busch et al., 2016; Sherman et al., 2011a) 41 % of patients undergoing left-sided temporal lobe resections demonstrated clinically meaningful declines in confrontation naming, compared to only 5 % of patients who had right-sided surgeries (Busch et al., 2016). In another study, based on the data extracted from the Swedish registry for epilepsy, hemiparesis after ATL was lower than 2 % (Bjellvi et al., 2014). According to the literature, asymptomatic homonymous contralateral quadrantanopia may occur in 15 %–90 % (Van et al., 2018), while symptomatic homonymous contralateral quadrantanopia was observed in 6 % of patients (Brotis et al., 2019). These findings further highlight the risks associated with these procedures. In a patient cohort published by Popovic et al. VFDs occurred in 21.5 % of adult patients (Popovic et al., 1995), while in a more recent cohort including solely pediatric patients, 22 % of them developed postoperative VFDs (Kim et al., 2008). It has to be taken into consideration that the reported incidence in the pertinent literature may well underestimate the actual VFD incidence. This is due to the variability in the definition of VFDs, differences in the used screening methods in the relevant studies, and in the inherent limitations of our current meta-analysis. The spectrum of VFDs varied widely in the literature, ranging from severe symptomatic homonymous quadrantanopia to mild, asymptomatic defects. Additionally, many surgeons consider VFDs as an expected side effect of the procedure rather than a complication, making the existent landscape more confusing and the drawing of any conclusions even more challenging. We consider VFD to be both an expected side effect and a complication of the surgery, as it potentially has impact on the patients' everyday activities (eg., driving ability). Therefore, systematic ophthalmologic assessment of the VFD after surgery is crucial. It has to be emphasized that the incidence of hemianopia is quite low, occurring in 1.2–1.3 % of the cases (Mathon et al., 2017). Although our panelists reached no consensus regarding the precise frequency of VFDs, the majority of them agreed that the complication rate exceeded 16 % among patients undergoing ATL. Conversely, selAH appeared to be associated with a lower incidence of VFDs, with panelists reporting rates of less than 2 % in pediatric patients, and between 5 and 10 % in adult patients. Furthermore, our panelists emphasized the potential role of MRI-based tractography for pre-operatively outlining the visual tracts, and particularly Meyer's loop, for a safer preoperative planning. Moreover, the potential employment of intra-operative cortical and subcortical stimulation techniques may improve surgical precision, and thus further reduce the postoperative occurrence of VFDs. Interestingly, Thudium et al. have reported their experience from applying DTI in their preoperative planning (Kim et al., 2008). Despite the usage of DTI in surgical planning, they still encountered a 25 % incidence of incomplete superior quadrantanopia in their series (Thudium et al., 2010). It is worth mentioning that in our consensus the panelists using systematic visual assessment have reported higher complication rates, suggesting underdetection in centers without such protocols or appropriate testing tools. This may be indicative of underreporting of post-operative VFDs in centers where there is no thorough pre- and post-operative evaluation. However, the higher detection rate of visual field defects (VFDs) following ATL or selAH in centers that perform both pre- and postoperative visual field testing is, in most cases, not clinically significant and does not result in functional limitations in daily life (Nandana et al., 2024).

Among the most frequently reported complications, post-operative cognitive, behavioral, and/or psychiatric disorders present significant challenges for the patients, their relatives, and the team of medical and paramedical caregivers. Post-operative psychiatric or behavioral disorders may be due to the exacerbation of non-detected, pre-existing disorders, or due to the development of de novo conditions. Previously published studies suggest that some forms of cognitive impairment arise in up to 7 % of cases (Brotis et al., 2019) while behavioral or psychiatric conditions, such as depression or anxiety, are reported in up to 18 % of cases (Macrodimitris et al., 2011). Our panelists underscored the need for comprehensive neuropsychological testing, assessing multiple cognitive domains, both before and after surgery, for developing an efficacious, individualized, rehabilitation plan. It has been previously reported that the NIH Common Data elements (CDEs) provide standardized neuropsychological test batteries such as Rey Auditory Learning test and Boston Naming Test for cognitive testing, clinical classifications and have been used for epilepsy research, including surgical outcome studies like ATL or selAH (Loring et al., 2011a). The adaptation and broad usage of such a battery will provide comparable data between different clinical series and a more accurate evaluation of the impact of ATL or selAH on the neurocognitive profile of these patients. Nonetheless, our panelists noted that no single neuropsychological assessment tool has gained universal acceptance. It is noteworthy to mention that if neurosurgeons are not referring for assessment patients of mood issues or anxiety prior to and following intervention, there may be lacking data to answer the question regarding which battery is better for psychological assessment. Similarly, while the literature and expert opinion lack clear representations for the specific rates of neuropsychiatric disorders following surgery, there is a consensus on the importance of using effective, multifaceted, assessment tools. It is important to note that those from our panelists, who routinely employed pre- and post-operative psychiatric evaluation in their patients, showed a higher incidence of psychiatric and behavioral changes after surgery.

In our current consensus memory outcome was not separately evaluated, since this was included in the cumulative neurocognitive outcome. Given that both ATL and selAH involve resection of medial temporal structures, particularly the hippocampus, memory outcomes are a crucial aspect of surgical evaluation and postoperative follow-up. Numerous studies have demonstrated that memory decline, particularly in verbal memory following dominant hemisphere resections, is a well-documented complication. For instance, Sherman et al. (2011) (Sherman et al., 2011b) reported that verbal memory deficits occur in up to 47 % of patients undergoing temporal lobectomy on the dominant hemisphere, while visual memory impairments are more common following nondominant resections. In contrast, more selective procedures such as selAH seem to be associated with relatively preserved memory functions. Drane et al. (2021) (Drane et al., 2021a) found that verbal memory decline occurred in only 14.3 % of patients undergoing selAH, compared to 47.4 % of those who had an ATL on the dominant hemisphere. These findings underscore the importance of comprehensive neuropsychological assessments pre- and post-operatively, with specific attention to domain-specific memory functions. Furthermore, the NIH Common Data Elements (CDEs) provide standardized guidelines for memory testing in epilepsy research, recommending tools such as the Rey Auditory Verbal Learning Test (RAVLT) and the Wechsler Memory Scale to ensure consistency and comparability across studies (Loring et al., 2011; NINDS, 2011) (Loring et al., 2011b)

A thorough meta-analysis by Tebo et al. (2014), examining the evolution of complication rates in patients undergoing ATL with or without AH, identifies neurological deficits, infections, and hemorrhages as some of the most commonly encountered postoperative complications, occurring in 19 %, 1.4 %, and 1.3 %, respectively. Other complications reported in the literature include cranial nerve deficits, particularly affecting the oculomotor and trochlear nerves (Jacobson et al., 1995), hydrocephalus, cerebrospinal fluid (CSF)-related complications, and extra-axial fluid collections (Georgiadis et al., 2013). In a recent retrospective case series by Dalio et al. (2022), it was observed that 86 % of patients undergoing ATL experienced no complications, although approximately 5 % of the cohort reported chronic, persistent headaches after surgery. Our panelists suggested that infection rates could be further reduced with the use of prophylactic antibiotics, particularly second-generation cephalosporins, which offer broader coverage. A subset of our panelists (25 %) also recommended avoiding shaving during the procedure, as this might help further reduce the infection risks. Regarding hematomas, our participants reported a low incidence, generally less than 5 %, and recommended meticulous intraoperative hemostasis for eliminating the need for routine wound drain placement, and mitigating the incidence of postoperative hematomas.

Recent advances in preoperative evaluation have emphasized the role of functional imaging in predicting cognitive outcomes. Binder et al. (2008) demonstrated that preoperative functional MRI (fMRI) could effectively predict verbal memory decline following left anterior temporal lobectomy (Binder et al., 2008). In a cohort of 60 patients, those with stronger preoperative memory, later epilepsy onset, and left-hemisphere language dominance on fMRI were at higher risk of significant postoperative memory loss. Notably, fMRI added predictive value beyond traditional measures like the Wada test, supporting its use as a noninvasive, clinically informative tool in surgical planning. These findings underscore the importance of incorporating advanced neuroimaging into patient-specific risk stratification strategies.

Notably, previous studies by Brotis et al. and Georgiadis et al. (Brotis et al., 2019; Georgiadis et al., 2013) highlight a significant decline in postoperative complication rates in this type of surgery since the 1980s. This reduction can be attributed to refinements of the employed surgical techniques, improved imaging techniques for more accurate and safer preoperative planning, and refined postoperative care strategies, which collectively contribute to improved safety and efficacy of temporal lobectomy procedures. The incorporation of modern imaging technologies, such as fMRI and diffusion-based imaging, and the adoption of more sophisticated neurosurgical tools and intraoperative electrophysiological monitoring techniques have played a crucial role in minimizing risks and improving surgical outcomes. Besides the technological advances, it is also critical to properly train young neurosurgeons in the field of epilepsy surgery. The best way to do this is by providing high-quality epilepsy surgery training through structured fellowships in high-volume centers, and epilepsy surgery educational hands-on courses and workshops (Rössler et al., 2024).

## 6 Study limitations

Several limitations characterize our present consensus. First, a key limitation is that the expert panel participated in the consensus process is relatively small in size and our survey was restricted to European centers. Furthermore, the panelists were selected from a list of hospitals in Europe with a recognized interest in epilepsy surgery, which carries the bias risk of excluding potentially significant experts with limited academic activities or lower publication rates. Second, the study relied on self-reported data from participants, which can be susceptible to social desirability bias, and/or other forms of response biases. Third, the absence of longitudinal data constitutes another limitation of our study. Our study's cross-sectional nature means that conclusions cannot be drawn regarding the long-term impacts or sustainability of the interventions. Fourth, in order to maintain a concise number of questions, our results were not overly stratified to specific populations, including the elderly, or those from middle- and low-income countries. Finally, our current study was unable to account for potential confounding factors related to the panelists, such as their levels of clinical experience, research expertise, or institutional affiliations. This was due to the responses being kept anonymous, which was intended to mitigate the influence of powerful voices and the suppression of dissent. In addition, all of the participants are epilepsy neurosurgeons and may underreport complications not routinely assessed by themselves for example visual deficits, cognitive changes, and/or psychiatric or neuropsychological outcomes. Due to the anonymous nature of the survey, it was not possible to stratify results by practice setting, years of experience, or surgical volume. This limits our ability to assess differences across practice environments. While this approach was aimed at ensuring an unbiased consensus, it also limited the ability to understand how individual characteristics might have shaped the final outcomes.

### 6.1 Emerging alternative treatments

Laser Interstitial Thermal Therapy (LITT) is a minimally invasive surgical technique, that has been employed in the management of DRE, including TLE, and represents an alternative treatment option (Hoppe et al., 2017; Shukla et al., 2017). It could be postulated that the minimally invasive character of LITT may be theoretically associated with fewer neurocognitive side effects and complications. Indeed, Guinle et al. (2023), in a series of pediatric patients with epilepsy of various etiology, found that 45.9 % of their cases demonstrated improvement of their neurocognitive profile after LITT treatment (Guinle et al., 2023). In addition, Gross et al. (2018) reported favorable seizure control, with a reduced cognitive burden in patients undergoing stereotactic laser amygdalohippocampotomy, particularly in those with mesial temporal lobe epilepsy (MTLE). (Gross et al., 2018). Furthermore, Drane et al. (2021) (Drane et al., 2021b) demonstrated that patients treated with stereotactic laser amygdalohippocampotomy in the language-dominant hemisphere had significantly better verbal memory outcomes compared to those who underwent open anterior temporal lobectomy (ATL). They reported that only 14.3 % of stereotactic laser amygdalohippocampotomy patients experienced verbal memory decline versus 47.4 % in the open surgery group. However, reported results across the literature remain controversial. For example, a recent metanalysis study by Alomar et al. revealed a decline of 24.2 % in verbal memory, and 25.2 % decline in visual memory among patients undergoing LITT for TLE. Likewise, naming ability showed a decline of 13.4 % in their meta-analysis. When these findings were compared to the existent literature data, it was noted that cognitive outcomes after LITT were somewhat less favorable than those following temporal lobectomy (Alomar et al., 2023). Another meta-analysis examined the efficacy of LITT in managing mTLE, finding that about 55 % of cases achieved seizure freedom. However, the effectiveness of LITT appears to decline over time (Brotis et al., 2021). Regarding the incidence of VFDs, it seems that this constitutes the most common neurological complication after LITT for epilepsy of mediotemporal origin (Jermakowicz et al., 2019). It becomes apparent that long-term follow up of LITT series is required for providing accurate seizure freedom data, as well as the incidence for VFD, and cognitive.

## 7 Conclusions

In conclusion, ATL and selAH represent effective surgical strategies for managing DRE of the temporal lobe. Despite the various risks associated with these procedures, including neurological deficits, infections, and psychiatric side effects, advancements in surgical techniques, careful preoperative planning, and enhanced postoperative care have significantly reduced complication rates since the 1980s. Continued research efforts and the implementation of comprehensive, multi-dimensional assessment tools together with the establishment of structured epilepsy surgery fellowships and the offering of adequate brain dissection courses remain essential to better train future epilepsy surgeons and thus further minimize procedure-associated risks, enhancing patient seizure and quality of life outcomes, and constantly refining treatment protocols.

## Disclosures

The authors have no personal, financial, or institutional interest in any of the drugs, materials, or devices described in this article.

## Funding

This research received no funding.

## Declaration of competing interest

The authors declare that they have no known competing financial interests or personal relationships that could have appeared to influence the work reported in this paper.
