# Supplementary material for: Anterior temporal lobectomy and selective AmygdaloHippocampectomy complications across Europe: review, meta-analysis, and Delphi consensus
Source: Brain Spine. 2025 Jun 18;5:104304. doi: 10.1016/j.bas.2025.104304 (PMC12272908; doi:10.1016/j.bas.2025.104304)
Supplement: Multimedia component 1 [file mmc1.docx]

**Supplementary Table 2**

## Frequencies

| Frequencies of 1. What is the observed mortality after _______? [ATL in children] | | | | | | | |
| --- | --- | --- | --- | --- | --- | --- | --- |
| **1. What is the observed mortality after _______?** | | **Counts** | | **% of Total** | | **Cumulative %** | |
| 0-2% |  | 40 |  | 100.0 % |  | 100.0 % |  |
|  | | | | | | | |

| Frequencies of 1. What is the observed mortality after _______? [selAH in children] | | | | | | | |
| --- | --- | --- | --- | --- | --- | --- | --- |
| **1. What is the observed mortality after _______?** | | **Counts** | | **% of Total** | | **Cumulative %** | |
| 0-2% |  | 30 |  | 100.0 % |  | 100.0 % |  |
|  | | | | | | | |

| Frequencies of 1. What is the observed mortality after _______? [ATL in adults] | | | | | | | |
| --- | --- | --- | --- | --- | --- | --- | --- |
| **1. What is the observed mortality after _______?** | | **Counts** | | **% of Total** | | **Cumulative %** | |
| 0-2% |  | 37 |  | 100.0 % |  | 100.0 % |  |
|  | | | | | | | |

| Frequencies of 1. What is the observed mortality after _______? [selAH in adults] | | | | | | | |
| --- | --- | --- | --- | --- | --- | --- | --- |
| **1. What is the observed mortality after _______?** | | **Counts** | | **% of Total** | | **Cumulative %** | |
| 0-2% |  | 31 |  | 100.0 % |  | 100.0 % |  |
|  | | | | | | | |

| Frequencies of 2. What is the occurrence rate of visual deficits after _______? [ATL in children] | | | | | | | |
| --- | --- | --- | --- | --- | --- | --- | --- |
| **2. What is the occurrence rate of visual deficits after _______?** | | **Counts** | | **% of Total** | | **Cumulative %** | |
| 0-2% |  | 4 |  | 10.0 % |  | 10.0 % |  |
| 11-15% |  | 5 |  | 12.5 % |  | 22.5 % |  |
| 3-5% |  | 8 |  | 20.0 % |  | 42.5 % |  |
| 6-10% |  | 11 |  | 27.5 % |  | 70.0 % |  |
| >16% |  | 12 |  | 30.0 % |  | 100.0 % |  |
|  | | | | | | | |

| Frequencies of 2. What is the occurrence rate of visual deficits after _______? [selAH in children] | | | | | | | |
| --- | --- | --- | --- | --- | --- | --- | --- |
| **2. What is the occurrence rate of visual deficits after _______?** | | **Counts** | | **% of Total** | | **Cumulative %** | |
| 0-2% |  | 8 |  | 30.8 % |  | 30.8 % |  |
| 11-15% |  | 4 |  | 15.4 % |  | 46.2 % |  |
| 3-5% |  | 4 |  | 15.4 % |  | 61.5 % |  |
| 6-10% |  | 6 |  | 23.1 % |  | 84.6 % |  |
| >16% |  | 4 |  | 15.4 % |  | 100.0 % |  |
|  | | | | | | | |

| Frequencies of 2. What is the occurrence rate of visual deficits after _______? [ATL in adults] | | | | | | | |
| --- | --- | --- | --- | --- | --- | --- | --- |
| **2. What is the occurrence rate of visual deficits after _______?** | | **Counts** | | **% of Total** | | **Cumulative %** | |
| 0-2% |  | 2 |  | 5.4 % |  | 5.4 % |  |
| 11-15% |  | 5 |  | 13.5 % |  | 18.9 % |  |
| 3-5% |  | 6 |  | 16.2 % |  | 35.1 % |  |
| 6-10% |  | 9 |  | 24.3 % |  | 59.5 % |  |
| >16% |  | 15 |  | 40.5 % |  | 100.0 % |  |
|  | | | | | | | |

| Frequencies of 2. What is the occurrence rate of visual deficits after _______? [selAH in adults] | | | | | | | |
| --- | --- | --- | --- | --- | --- | --- | --- |
| **2. What is the occurrence rate of visual deficits after _______?** | | **Counts** | | **% of Total** | | **Cumulative %** | |
| 0-2% |  | 7 |  | 25.0 % |  | 25.0 % |  |
| 11-15% |  | 5 |  | 17.9 % |  | 42.9 % |  |
| 3-5% |  | 3 |  | 10.7 % |  | 53.6 % |  |
| 6-10% |  | 8 |  | 28.6 % |  | 82.1 % |  |
| >16% |  | 5 |  | 17.9 % |  | 100.0 % |  |
|  | | | | | | | |

| Frequencies of 3. Which of the following measures would you consider to avoid visual field deficits? | | | | | | | |
| --- | --- | --- | --- | --- | --- | --- | --- |
| **3. Which of the following measures would you consider to avoid visual field deficits?** | | **Counts** | | **% of Total** | | **Cumulative %** | |
| Intra-operative stimulation for identification of visual pathway |  | 4 |  | 10.3 % |  | 10.3 % |  |
| Pre-op DTI for identification of visual pathway |  | 21 |  | 53.8 % |  | 64.1 % |  |
| Pre-op DTI for identification of visual pathway;Intraoperative visual evoked potentials (VEPs) |  | 1 |  | 2.6 % |  | 66.7 % |  |
| Pre-op DTI for identification of visual pathway;Intraoperative visual evoked potentials (VEPs);Intra-operative stimulation for identification of visual pathway |  | 3 |  | 7.7 % |  | 74.4 % |  |
| Pre-op DTI for identification of visual pathway;Precise preparation along the intraoperative anatomy, if necessary using intraoperative neuronavigation |  | 1 |  | 2.6 % |  | 76.9 % |  |
| Pre-op DTI for identification of visual pathway;basal approaches |  | 1 |  | 2.6 % |  | 79.5 % |  |
| Pre-op DTI for identification of visual pathway;generally basal approaches |  | 1 |  | 2.6 % |  | 82.1 % |  |
| Pre-op DTI for identification of visual pathway;neuronavigation with visual field DTIs intraoperatively |  | 1 |  | 2.6 % |  | 84.6 % |  |
| Pre-op DTI for identification of visual pathway;posteriorly very limited neocortical resection |  | 1 |  | 2.6 % |  | 87.2 % |  |
| The methods currently available are not accurate enough to be proposed in the surgical setting |  | 1 |  | 2.6 % |  | 89.7 % |  |
| anatomical considerations. |  | 1 |  | 2.6 % |  | 92.3 % |  |
| limited resection |  | 1 |  | 2.6 % |  | 94.9 % |  |
| relationship between the posterior third of the hippocampus body - tail and the area of the Meyer loop |  | 1 |  | 2.6 % |  | 97.4 % |  |
| supracerebellar transtentorial approach for AHE |  | 1 |  | 2.6 % |  | 100.0 % |  |
|  | | | | | | | |

| Frequencies of 4. How would you detect visual deficits in your service routinely? | | | | | | | |
| --- | --- | --- | --- | --- | --- | --- | --- |
| **4. How would you detect visual deficits in your service routinely?** | | **Counts** | | **% of Total** | | **Cumulative %** | |
| Clinical examination |  | 12 |  | 29.3 % |  | 29.3 % |  |
| I don't routinely examine visual fields |  | 2 |  | 4.9 % |  | 34.1 % |  |
| Visual field examination |  | 27 |  | 65.9 % |  | 100.0 % |  |
|  | | | | | | | |

| Frequencies of 5. What is the observed frequency rate of permanent motor deficits rate after _______? [ATL in children] | | | | | | | |
| --- | --- | --- | --- | --- | --- | --- | --- |
| **5. What is the observed frequency rate of permanent motor deficits rate after _______?** | | **Counts** | | **% of Total** | | **Cumulative %** | |
| 0-2% |  | 37 |  | 92.5 % |  | 92.5 % |  |
| 3-5% |  | 3 |  | 7.5 % |  | 100.0 % |  |
|  | | | | | | | |

| Frequencies of 5. What is the observed frequency rate of permanent motor deficits rate after _______? [selAH in children] | | | | | | | |
| --- | --- | --- | --- | --- | --- | --- | --- |
| **5. What is the observed frequency rate of permanent motor deficits rate after _______?** | | **Counts** | | **% of Total** | | **Cumulative %** | |
| 0-2% |  | 26 |  | 100.0 % |  | 100.0 % |  |
|  | | | | | | | |

| Frequencies of 5. What is the observed frequency rate of permanent motor deficits rate after _______? [ATL in adults] | | | | | | | |
| --- | --- | --- | --- | --- | --- | --- | --- |
| **5. What is the observed frequency rate of permanent motor deficits rate after _______?** | | **Counts** | | **% of Total** | | **Cumulative %** | |
| 0-2% |  | 33 |  | 89.2 % |  | 89.2 % |  |
| 3-5% |  | 3 |  | 8.1 % |  | 97.3 % |  |
| 6-10% |  | 1 |  | 2.7 % |  | 100.0 % |  |
|  | | | | | | | |

| Frequencies of 5. What is the observed frequency rate of permanent motor deficits rate after _______? [selAH in adults] | | | | | | | |
| --- | --- | --- | --- | --- | --- | --- | --- |
| **5. What is the observed frequency rate of permanent motor deficits rate after _______?** | | **Counts** | | **% of Total** | | **Cumulative %** | |
| 0-2% |  | 23 |  | 85.2 % |  | 85.2 % |  |
| 3-5% |  | 4 |  | 14.8 % |  | 100.0 % |  |
|  | | | | | | | |

| Frequencies of 6. Which of the following measures would you routinely routinely to avoid motor deficits? | | | | | | | |
| --- | --- | --- | --- | --- | --- | --- | --- |
| **6. Which of the following measures would you routinely routinely to avoid motor deficits?** | | **Counts** | | **% of Total** | | **Cumulative %** | |
| All, especially the last option |  | 1 |  | 2.8 % |  | 2.8 % |  |
| Anatomical landmarks (choroidal plexus, perforating arteries) |  | 1 |  | 2.8 % |  | 5.6 % |  |
| Avoid Transsylvian approaches. |  | 1 |  | 2.8 % |  | 8.3 % |  |
| Direct cortical and subcortical stimulation |  | 5 |  | 13.9 % |  | 22.2 % |  |
| I don't use any of the above for patients who are undergoing ATL. However for eloquent region surgery, I use all of them. |  | 1 |  | 2.8 % |  | 25.0 % |  |
| In general, however i don’t do it for AMTL |  | 1 |  | 2.8 % |  | 27.8 % |  |
| MEPs, ESSPs |  | 5 |  | 13.9 % |  | 41.7 % |  |
| Motor deficit are rare during ATL (nearly 0%) so that I do not consider IOM |  | 1 |  | 2.8 % |  | 44.4 % |  |
| No test necessary. |  | 1 |  | 2.8 % |  | 47.2 % |  |
| None |  | 6 |  | 16.7 % |  | 63.9 % |  |
| Pre-op fMRI |  | 3 |  | 8.3 % |  | 72.2 % |  |
| advanced dissection technique |  | 1 |  | 2.8 % |  | 75.0 % |  |
| anatomy. ATL is an anatomical procedure, and no need for motor pathway involvment |  | 1 |  | 2.8 % |  | 77.8 % |  |
| avoid mesial coagulation |  | 1 |  | 2.8 % |  | 80.6 % |  |
| avoidance of mesial coagulation |  | 1 |  | 2.8 % |  | 83.3 % |  |
| from my point of view, motor deficits after temporal resections in epilepsy patients (both SAH and ATL) only occur as a consequence of a complication (e.g. postoperative bleeding) and are therefore extremely rare and not result of the intraoperative strategy or monitoring sitation. i therefore think that intraoperative monitoring would not change this, as a motor deficit should not be due to the intraoperative technique / extent of resection. In my opinion, however, intraoperative monitoring with MEPs, subcortical stimulation etc. is indispensable in the case of glial tumors and/or involvement of extratemporal structures (insula etc.) |  | 1 |  | 2.8 % |  | 86.1 % |  |
| none |  | 2 |  | 5.6 % |  | 91.7 % |  |
| none |  | 1 |  | 2.8 % |  | 94.4 % |  |
| none of above, more important good microsurgical technique and intraoperative navigation |  | 1 |  | 2.8 % |  | 97.2 % |  |
| nothing |  | 1 |  | 2.8 % |  | 100.0 % |  |
|  | | | | | | | |

| Frequencies of 7. Would you routinely employ TCD to detect postoperative vasospasm? | | | | | | | |
| --- | --- | --- | --- | --- | --- | --- | --- |
| **7. Would you routinely employ TCD to detect postoperative vasospasm?** | | **Counts** | | **% of Total** | | **Cumulative %** | |
| No |  | 40 |  | 97.6 % |  | 97.6 % |  |
| Yes |  | 1 |  | 2.4 % |  | 100.0 % |  |
|  | | | | | | | |

| Frequencies of 8. What is the observed early (<30 days) infection rate after _______? [ATL in children] | | | | | | | |
| --- | --- | --- | --- | --- | --- | --- | --- |
| **8. What is the observed early (<30 days) infection rate after _______?** | | **Counts** | | **% of Total** | | **Cumulative %** | |
| 0-2% |  | 37 |  | 92.5 % |  | 92.5 % |  |
| 3-5% |  | 2 |  | 5.0 % |  | 97.5 % |  |
| 6-10% |  | 1 |  | 2.5 % |  | 100.0 % |  |
|  | | | | | | | |

| Frequencies of 8. What is the observed early (<30 days) infection rate after _______? [selAH in children] | | | | | | | |
| --- | --- | --- | --- | --- | --- | --- | --- |
| **8. What is the observed early (<30 days) infection rate after _______?** | | **Counts** | | **% of Total** | | **Cumulative %** | |
| 0-2% |  | 25 |  | 96.2 % |  | 96.2 % |  |
| 3-5% |  | 1 |  | 3.8 % |  | 100.0 % |  |
|  | | | | | | | |

| Frequencies of 8. What is the observed early (<30 days) infection rate after _______? [ATL in adults] | | | | | | | |
| --- | --- | --- | --- | --- | --- | --- | --- |
| **8. What is the observed early (<30 days) infection rate after _______?** | | **Counts** | | **% of Total** | | **Cumulative %** | |
| 0-2% |  | 29 |  | 78.4 % |  | 78.4 % |  |
| 11-15% |  | 1 |  | 2.7 % |  | 81.1 % |  |
| 3-5% |  | 7 |  | 18.9 % |  | 100.0 % |  |
|  | | | | | | | |

| Frequencies of 8. What is the observed early (<30 days) infection rate after _______? [selAH in adults] | | | | | | | |
| --- | --- | --- | --- | --- | --- | --- | --- |
| **8. What is the observed early (<30 days) infection rate after _______?** | | **Counts** | | **% of Total** | | **Cumulative %** | |
| 0-2% |  | 20 |  | 71.4 % |  | 71.4 % |  |
| 3-5% |  | 7 |  | 25.0 % |  | 96.4 % |  |
| 6-10% |  | 1 |  | 3.6 % |  | 100.0 % |  |
|  | | | | | | | |

| Frequencies of 9. What is the late (<30 days) observed infection rate after _______? [ATL in children] | | | | | | | |
| --- | --- | --- | --- | --- | --- | --- | --- |
| **9. What is the late (<30 days) observed infection rate after _______?** | | **Counts** | | **% of Total** | | **Cumulative %** | |
| 0-2% |  | 38 |  | 97.4 % |  | 97.4 % |  |
| 3-5% |  | 1 |  | 2.6 % |  | 100.0 % |  |
|  | | | | | | | |

| Frequencies of 9. What is the late (<30 days) observed infection rate after _______? [selAH in children] | | | | | | | |
| --- | --- | --- | --- | --- | --- | --- | --- |
| **9. What is the late (<30 days) observed infection rate after _______?** | | **Counts** | | **% of Total** | | **Cumulative %** | |
| 0-2% |  | 24 |  | 96.0 % |  | 96.0 % |  |
| 3-5% |  | 1 |  | 4.0 % |  | 100.0 % |  |
|  | | | | | | | |

| Frequencies of 9. What is the late (<30 days) observed infection rate after _______? [ATL in adults] | | | | | | | |
| --- | --- | --- | --- | --- | --- | --- | --- |
| **9. What is the late (<30 days) observed infection rate after _______?** | | **Counts** | | **% of Total** | | **Cumulative %** | |
| 0-2% |  | 34 |  | 94.4 % |  | 94.4 % |  |
| 3-5% |  | 1 |  | 2.8 % |  | 97.2 % |  |
| 6-10% |  | 1 |  | 2.8 % |  | 100.0 % |  |
|  | | | | | | | |

| Frequencies of 9. What is the late (<30 days) observed infection rate after _______? [selAH in adults] | | | | | | | |
| --- | --- | --- | --- | --- | --- | --- | --- |
| **9. What is the late (<30 days) observed infection rate after _______?** | | **Counts** | | **% of Total** | | **Cumulative %** | |
| 0-2% |  | 24 |  | 88.9 % |  | 88.9 % |  |
| 3-5% |  | 3 |  | 11.1 % |  | 100.0 % |  |
|  | | | | | | | |

| Frequencies of 10. Do you shave hairs before surgery? | | | | | | | |
| --- | --- | --- | --- | --- | --- | --- | --- |
| **10. Do you shave hairs before surgery?** | | **Counts** | | **% of Total** | | **Cumulative %** | |
| No |  | 9 |  | 22.5 % |  | 22.5 % |  |
| Sometimes |  | 4 |  | 10.0 % |  | 32.5 % |  |
| Yes |  | 27 |  | 67.5 % |  | 100.0 % |  |
|  | | | | | | | |

| Frequencies of 11. Please describe your strategy on antibiotic prophylaxis for temporal lobectomy. | | | | | | | |
| --- | --- | --- | --- | --- | --- | --- | --- |
| **11. Please describe your strategy on antibiotic prophylaxis for temporal lobectomy.** | | **Counts** | | **% of Total** | | **Cumulative %** | |
| 24 Hhr cefazolin |  | 1 |  | 2.4 % |  | 2.4 % |  |
| 24h ATB |  | 1 |  | 2.4 % |  | 4.9 % |  |
| 48 hours |  | 1 |  | 2.4 % |  | 7.3 % |  |
| After induction of anesthesia before the skin incision iv 500mg or 1g cefazolin to pediatric and adult patients, respectively. |  | 1 |  | 2.4 % |  | 9.8 % |  |
| Cefacolin 30mg/kg before an 4 after the first dose |  | 1 |  | 2.4 % |  | 12.2 % |  |
| Cefalosporin and vancomycin started on the day of surgery before skin incision and continued for at least 7 and 2 days respectively. |  | 1 |  | 2.4 % |  | 14.6 % |  |
| Cefazolin (+/- Metronidazole if opening of mastoid air cells) |  | 1 |  | 2.4 % |  | 17.1 % |  |
| Cefazolin 2gx2 on the surgery day |  | 1 |  | 2.4 % |  | 19.5 % |  |
| Cefazolin 500/1000 mg iv at the time of incision to pediadric and adult patients, respectively |  | 1 |  | 2.4 % |  | 22.0 % |  |
| Cefazolin al least 30’ before incision |  | 1 |  | 2.4 % |  | 24.4 % |  |
| Cefazolin at least 30' before incision |  | 1 |  | 2.4 % |  | 26.8 % |  |
| Cefazolin at least 30’ before incision |  | 1 |  | 2.4 % |  | 29.3 % |  |
| Cefazolin at least 30’ before incision. Then repeated if needed |  | 1 |  | 2.4 % |  | 31.7 % |  |
| Cefazolin or Vancomycin in case of allergy, intra-operative every 4h and during the first 24h post-op. |  | 1 |  | 2.4 % |  | 34.1 % |  |
| Ceftriaxone 50mg/kg 1 hour before skin incision |  | 1 |  | 2.4 % |  | 36.6 % |  |
| Cefuroxim 1500 mg for adults, 50 mg/kg, max 1500 mg for children. Administered 30 min preop, repeated after 4 hours if eligible. In case of allergy, klindamycin 600 mg (30 mg/kg in children) is given. |  | 1 |  | 2.4 % |  | 39.0 % |  |
| Cephalosporin IV 30 mn before incision |  | 1 |  | 2.4 % |  | 41.5 % |  |
| Cephalosporin single shot |  | 1 |  | 2.4 % |  | 43.9 % |  |
| Cephazolin 1 h before, at 4 and at 12h |  | 1 |  | 2.4 % |  | 46.3 % |  |
| III generation Cephalosporin the day of surgery and the next two days |  | 1 |  | 2.4 % |  | 48.8 % |  |
| III generation cephalosporin (usually Ceftazidime) just before the skin incision and 72hrs (1g i.v every 12 hrs) |  | 1 |  | 2.4 % |  | 51.2 % |  |
| Intraoperative cefazolin |  | 1 |  | 2.4 % |  | 53.7 % |  |
| One shot therapy during surgery |  | 1 |  | 2.4 % |  | 56.1 % |  |
| Preop cefuroxim, 1.5 g for adults, 50 mg/kg max 1.5 g, administered 30 min preop and repeated after 4 hours if needed. |  | 1 |  | 2.4 % |  | 58.5 % |  |
| Single shot appication of antibiotics before skin incision |  | 1 |  | 2.4 % |  | 61.0 % |  |
| Unacid or cefazolin |  | 1 |  | 2.4 % |  | 63.4 % |  |
| We routinely use antibiotic prophylaxis for 48 hours, starting from the day of surgery |  | 1 |  | 2.4 % |  | 65.9 % |  |
| cefazolin 2gr i.v. 30 min prior skin incision |  | 1 |  | 2.4 % |  | 68.3 % |  |
| cefazoline 2gr preop iv and 3dd 1gr cefazoline during 24h postop |  | 1 |  | 2.4 % |  | 70.7 % |  |
| ceftriaxone |  | 1 |  | 2.4 % |  | 73.2 % |  |
| one dose cefuroxime |  | 1 |  | 2.4 % |  | 75.6 % |  |
| peds - vanco ceftriaxone before surg and for 24h. adults - Cefuroxime before incision only |  | 1 |  | 2.4 % |  | 78.0 % |  |
| preop 3g Ampicillin and Sulbactam |  | 1 |  | 2.4 % |  | 80.5 % |  |
| preoperative Cefuroxim |  | 1 |  | 2.4 % |  | 82.9 % |  |
| singel shot prior shin incision, cefuroxime or similar |  | 1 |  | 2.4 % |  | 85.4 % |  |
| single shot |  | 2 |  | 4.9 % |  | 90.2 % |  |
| single shot broad sprectrum Abx |  | 1 |  | 2.4 % |  | 92.7 % |  |
| single shot prophylaxis (cefuroxim of alternative) prior skin incision |  | 1 |  | 2.4 % |  | 95.1 % |  |
| single shot with cefuroxime |  | 1 |  | 2.4 % |  | 97.6 % |  |
| standard intraoperative antibiotic prophylaxis the latest 30min before incision, with a 24h protection, usually covering a large spectrum with a 3rd generation cephalosporin via i.v. route |  | 1 |  | 2.4 % |  | 100.0 % |  |
|  | | | | | | | |

| Frequencies of 12. The duration of surgery has ------------ on the incidence of postoperative infections. Choose the appropriate option to fill the gap. | | | | | | | |
| --- | --- | --- | --- | --- | --- | --- | --- |
| **12. The duration of surgery has ------------ on the incidence of postoperative infections. Choose the appropriate option to fill the gap.** | | **Counts** | | **% of Total** | | **Cumulative %** | |
| a negative impact |  | 15 |  | 36.6 % |  | 36.6 % |  |
| a positive impact |  | 2 |  | 4.9 % |  | 41.5 % |  |
| no role |  | 21 |  | 51.2 % |  | 92.7 % |  |
| not clear |  | 1 |  | 2.4 % |  | 95.1 % |  |
| only in extreme cases |  | 1 |  | 2.4 % |  | 97.6 % |  |
| probably a mild negative impact. i think the extent of the skin and bone flap has the most impact (literature supporting that) and patient characteristics such as diabetes, history of wound infections e.g. |  | 1 |  | 2.4 % |  | 100.0 % |  |
|  | | | | | | | |

| Frequencies of 13. What is the observed occurrence rate of postoperative hematomas requiring surgical management? [In children after ATL] | | | | | | | |
| --- | --- | --- | --- | --- | --- | --- | --- |
| **13. What is the observed occurrence rate of postoperative hematomas requiring surgical management?** | | **Counts** | | **% of Total** | | **Cumulative %** | |
| 0-5% |  | 39 |  | 97.5 % |  | 97.5 % |  |
| 6-10% |  | 1 |  | 2.5 % |  | 100.0 % |  |
|  | | | | | | | |

| Frequencies of 13. What is the observed occurrence rate of postoperative hematomas requiring surgical management? [In children after selAH] | | | | | | | |
| --- | --- | --- | --- | --- | --- | --- | --- |
| **13. What is the observed occurrence rate of postoperative hematomas requiring surgical management?** | | **Counts** | | **% of Total** | | **Cumulative %** | |
| 0-5% |  | 26 |  | 100.0 % |  | 100.0 % |  |
|  | | | | | | | |

| Frequencies of 13. What is the observed occurrence rate of postoperative hematomas requiring surgical management? [In adults after ATL] | | | | | | | |
| --- | --- | --- | --- | --- | --- | --- | --- |
| **13. What is the observed occurrence rate of postoperative hematomas requiring surgical management?** | | **Counts** | | **% of Total** | | **Cumulative %** | |
| 0-5% |  | 37 |  | 100.0 % |  | 100.0 % |  |
|  | | | | | | | |

| Frequencies of 13. What is the observed occurrence rate of postoperative hematomas requiring surgical management? [In adults after selAH] | | | | | | | |
| --- | --- | --- | --- | --- | --- | --- | --- |
| **13. What is the observed occurrence rate of postoperative hematomas requiring surgical management?** | | **Counts** | | **% of Total** | | **Cumulative %** | |
| 0-5% |  | 28 |  | 100.0 % |  | 100.0 % |  |
|  | | | | | | | |

| Frequencies of 14. Would you routinely use a drain to avoid postoperative hematomas? | | | | | | | |
| --- | --- | --- | --- | --- | --- | --- | --- |
| **14. Would you routinely use a drain to avoid postoperative hematomas?** | | **Counts** | | **% of Total** | | **Cumulative %** | |
| No |  | 35 |  | 85.4 % |  | 85.4 % |  |
| Some times |  | 5 |  | 12.2 % |  | 97.6 % |  |
| Yes |  | 1 |  | 2.4 % |  | 100.0 % |  |
|  | | | | | | | |

| Frequencies of 15. What is the frequency of psychiatric manifestations after _______? [ATL in children] | | | | | | | |
| --- | --- | --- | --- | --- | --- | --- | --- |
| **15. What is the frequency of psychiatric manifestations after _______?** | | **Counts** | | **% of Total** | | **Cumulative %** | |
| 0-2% |  | 29 |  | 72.5 % |  | 72.5 % |  |
| 3-5% |  | 10 |  | 25.0 % |  | 97.5 % |  |
| 6-10% |  | 1 |  | 2.5 % |  | 100.0 % |  |
|  | | | | | | | |

| Frequencies of 15. What is the frequency of psychiatric manifestations after _______? [selAH in children] | | | | | | | |
| --- | --- | --- | --- | --- | --- | --- | --- |
| **15. What is the frequency of psychiatric manifestations after _______?** | | **Counts** | | **% of Total** | | **Cumulative %** | |
| 0-2% |  | 22 |  | 84.6 % |  | 84.6 % |  |
| 3-5% |  | 4 |  | 15.4 % |  | 100.0 % |  |
|  | | | | | | | |

| Frequencies of 15. What is the frequency of psychiatric manifestations after _______? [ATL in adults] | | | | | | | |
| --- | --- | --- | --- | --- | --- | --- | --- |
| **15. What is the frequency of psychiatric manifestations after _______?** | | **Counts** | | **% of Total** | | **Cumulative %** | |
| 0-2% |  | 13 |  | 36.1 % |  | 36.1 % |  |
| 11-15% |  | 4 |  | 11.1 % |  | 47.2 % |  |
| 16-20% |  | 1 |  | 2.8 % |  | 50.0 % |  |
| 3-5% |  | 13 |  | 36.1 % |  | 86.1 % |  |
| 6-10% |  | 5 |  | 13.9 % |  | 100.0 % |  |
|  | | | | | | | |

| Frequencies of 15. What is the frequency of psychiatric manifestations after _______? [selAH in adults] | | | | | | | |
| --- | --- | --- | --- | --- | --- | --- | --- |
| **15. What is the frequency of psychiatric manifestations after _______?** | | **Counts** | | **% of Total** | | **Cumulative %** | |
| 0-2% |  | 12 |  | 44.4 % |  | 44.4 % |  |
| 11-15% |  | 3 |  | 11.1 % |  | 55.6 % |  |
| 3-5% |  | 8 |  | 29.6 % |  | 85.2 % |  |
| 6-10% |  | 4 |  | 14.8 % |  | 100.0 % |  |
|  | | | | | | | |

| Frequencies of 16. Would you routinely employ pre- and postoperative psychiatric evaluation after temporal lobectomy? | | | | | | | |
| --- | --- | --- | --- | --- | --- | --- | --- |
| **16. Would you routinely employ pre- and postoperative psychiatric evaluation after temporal lobectomy?** | | **Counts** | | **% of Total** | | **Cumulative %** | |
| No |  | 22 |  | 53.7 % |  | 53.7 % |  |
| Other |  | 1 |  | 2.4 % |  | 56.1 % |  |
| Some times |  | 12 |  | 29.3 % |  | 85.4 % |  |
| Yes |  | 6 |  | 14.6 % |  | 100.0 % |  |
|  | | | | | | | |

| Frequencies of 17. If you answered "Yes" in the previous question, please specify which tests or batteries would you employ? | | | | | | | |
| --- | --- | --- | --- | --- | --- | --- | --- |
| **17. If you answered "Yes" in the previous question, please specify which tests or batteries would you employ?** | | **Counts** | | **% of Total** | | **Cumulative %** | |
| Madras, BDI, BPRS, Hamilton anxiety and depression, temps-a and MCMI II |  | 1 |  | 12.5 % |  | 12.5 % |  |
| BDI, STAI S & T, QOLIE-31, SDS, M.I.N.I., BPRS. |  | 1 |  | 12.5 % |  | 25.0 % |  |
| Hamilton |  | 1 |  | 12.5 % |  | 37.5 % |  |
| Looking for psychiatric symptoms and well beeing, Changes since surgery, Psychological status testing |  | 1 |  | 12.5 % |  | 50.0 % |  |
| consult with psychiatrist |  | 1 |  | 12.5 % |  | 62.5 % |  |
| extensive neuropsychological evaluation including depression scales (e.g. BDI), executive functions, attention. Evaluation of preexisting psychiatric co-morbidities (quite high in adult epilepsy patients) |  | 1 |  | 12.5 % |  | 75.0 % |  |
| neuropsychological evaluation not psychiatric |  | 1 |  | 12.5 % |  | 87.5 % |  |
| tests are tailored accoring to patients psychiatric profile, previous frequencies of postop psychiatric manifestations are transient |  | 1 |  | 12.5 % |  | 100.0 % |  |
|  | | | | | | | |

| Frequencies of 18. What is the observed frequency of cognitive manifestations after _______? [ATL in children] | | | | | | | |
| --- | --- | --- | --- | --- | --- | --- | --- |
| **18. What is the observed frequency of cognitive manifestations after _______?** | | **Counts** | | **% of Total** | | **Cumulative %** | |
| 0-2% |  | 18 |  | 46.2 % |  | 46.2 % |  |
| 11-15% |  | 2 |  | 5.1 % |  | 51.3 % |  |
| 16-20% |  | 1 |  | 2.6 % |  | 53.8 % |  |
| 3-5% |  | 14 |  | 35.9 % |  | 89.7 % |  |
| 6-10% |  | 4 |  | 10.3 % |  | 100.0 % |  |
|  | | | | | | | |

| Frequencies of 18. What is the observed frequency of cognitive manifestations after _______? [selAH in children] | | | | | | | |
| --- | --- | --- | --- | --- | --- | --- | --- |
| **18. What is the observed frequency of cognitive manifestations after _______?** | | **Counts** | | **% of Total** | | **Cumulative %** | |
| 0-2% |  | 14 |  | 53.8 % |  | 53.8 % |  |
| 11-15% |  | 1 |  | 3.8 % |  | 57.7 % |  |
| 16-20% |  | 1 |  | 3.8 % |  | 61.5 % |  |
| 3-5% |  | 8 |  | 30.8 % |  | 92.3 % |  |
| 6-10% |  | 2 |  | 7.7 % |  | 100.0 % |  |
|  | | | | | | | |

| Frequencies of 18. What is the observed frequency of cognitive manifestations after _______? [ATL in adults] | | | | | | | |
| --- | --- | --- | --- | --- | --- | --- | --- |
| **18. What is the observed frequency of cognitive manifestations after _______?** | | **Counts** | | **% of Total** | | **Cumulative %** | |
| 0-2% |  | 8 |  | 21.6 % |  | 21.6 % |  |
| 11-15% |  | 5 |  | 13.5 % |  | 35.1 % |  |
| 16-20% |  | 6 |  | 16.2 % |  | 51.4 % |  |
| 3-5% |  | 13 |  | 35.1 % |  | 86.5 % |  |
| 6-10% |  | 5 |  | 13.5 % |  | 100.0 % |  |
|  | | | | | | | |

| Frequencies of 18. What is the observed frequency of cognitive manifestations after _______? [selAH in adults] | | | | | | | |
| --- | --- | --- | --- | --- | --- | --- | --- |
| **18. What is the observed frequency of cognitive manifestations after _______?** | | **Counts** | | **% of Total** | | **Cumulative %** | |
| 0-2% |  | 9 |  | 32.1 % |  | 32.1 % |  |
| 11-15% |  | 3 |  | 10.7 % |  | 42.9 % |  |
| 16-20% |  | 5 |  | 17.9 % |  | 60.7 % |  |
| 3-5% |  | 5 |  | 17.9 % |  | 78.6 % |  |
| 6-10% |  | 6 |  | 21.4 % |  | 100.0 % |  |
|  | | | | | | | |

| Frequencies of 19. Would you routinely employ pre- and postoperative neuropsychological examinations after lobectomy? | | | | | | | |
| --- | --- | --- | --- | --- | --- | --- | --- |
| **19. Would you routinely employ pre- and postoperative neuropsychological examinations after lobectomy?** | | **Counts** | | **% of Total** | | **Cumulative %** | |
| No |  | 2 |  | 5.0 % |  | 5.0 % |  |
| Some times |  | 6 |  | 15.0 % |  | 20.0 % |  |
| Yes |  | 32 |  | 80.0 % |  | 100.0 % |  |
|  | | | | | | | |

| Frequencies of 20. If you answered yes in the previous question, please specify which tests or batteries of tests would you employ? | | | | | | | |
| --- | --- | --- | --- | --- | --- | --- | --- |
| **20. If you answered yes in the previous question, please specify which tests or batteries of tests would you employ?** | | **Counts** | | **% of Total** | | **Cumulative %** | |
| A complete neuropsychological evaluation is done including measures of Intellectual ability (Wechsler scales), verbal and visual memory (TAVECI / CALVT and Nepsy-II "memory for faces"), executive functions (Inhibition subtest from Nepsy-II), learning abilities (reading and writing), Visoconstruction (VMI, Rey complex figure...) and behaviour (including questionaries like Achenbach, Brief to parents and teachers). |  | 1 |  | 3.6 % |  | 3.6 % |  |
| BLAD (Verbal naming and repetition, reading, calculation or WAIS aritmetic, Orientation) A`s identification attentional test, trail making test A and B, TP, WMS-III, stroop color test, Rey perception, attentional and memory test, Raven Ab test. |  | 1 |  | 3.6 % |  | 7.1 % |  |
| Dedicated batteries recognized by the Italian League Against Epilepsy |  | 1 |  | 3.6 % |  | 10.7 % |  |
| Dedicated batteries validated by the Italian League Against Epilepsy |  | 1 |  | 3.6 % |  | 14.3 % |  |
| Dedicated batteries, validated by the Italian League Against Epilepsy |  | 1 |  | 3.6 % |  | 17.9 % |  |
| Dedicated testing which are validated by Italian League Against Epilepsy |  | 1 |  | 3.6 % |  | 21.4 % |  |
| Edinburgh hand dominance , WMS- R orientation, WMS-R semantic, WMS-V basic attention, WMS-III , Stroop test,Verbal fluency, Luria test, Clock drawing, WAIS, CAVLT , Boston naming, Mayo clinic speech function test, Benton face recognition, Hooper visual organisation, Rey osterrieth, figure copying, Navoln letters, Beck depression |  | 1 |  | 3.6 % |  | 25.0 % |  |
| Edinburgh handedness inventory, WMS, Stroop, WAIS, CAVLT, Rey Osterrieth, Boston naming test, mayo clinic speech language function exam, Benton face recognition, Hooper visual organization test, Albert's test, line bisection, figure copying, |  | 1 |  | 3.6 % |  | 28.6 % |  |
| Every patient selected for epilepsy surgery gets fully neuropsychological tested pre - and postoperatively |  | 1 |  | 3.6 % |  | 32.1 % |  |
| FWIT out of the D-KEFS=Delis/Kaplan Executive Function System; TMT=Trail Making Test; VLMT; DCSII; alertness-tests; WAIS-IV=Wechsler Adult Intelligence Scale – Fourth Edition; WMS-R=Wechsler memory test |  | 1 |  | 3.6 % |  | 35.7 % |  |
| IQ measures (which depends on age and developmental level - wppsi, wisc or bayley), fine motor (pegs, fingertip, grip strength etc), Visuo-motor integration (berry VMI), and parental reports of attention and executive functioning (BRIEF) and adaptive functioning (ABAS or Vineland). Maybe additional language test (which one depends on age and developmental level). |  | 1 |  | 3.6 % |  | 39.3 % |  |
| IQ, memory evaluations, attention evaluations |  | 1 |  | 3.6 % |  | 42.9 % |  |
| SWAIS-IV, MWT-B, TAP, Corsi, DCS, DMS 48, VLMT-A, Rey, Labyrinth, M-WCST, TMT, BDI II, SCL-90-S |  | 1 |  | 3.6 % |  | 46.4 % |  |
| WAIS, BNT, WMS III,BVMT-R, Grooved Pegboard, MFS, HADS,Verbal Flow and CWIT, Token Test,D-KEFS TMT, Rey Complex Figure Copying, Claeson-Dahl auditive learning test, |  | 1 |  | 3.6 % |  | 50.0 % |  |
| WISC IV, Child Behavior Checklist for Ages 6-18, (Achenbach e Rescorla, 2001), Quality of life (PEDSQL) |  | 1 |  | 3.6 % |  | 53.6 % |  |
| WISC IV, IQ, Child Behavior Checklist for Ages 6-18, PEDSQL |  | 1 |  | 3.6 % |  | 57.1 % |  |
| WISC, QI, Griffith, MMIT |  | 1 |  | 3.6 % |  | 60.7 % |  |
| WISC4, WPPSI3, NEPSY2, WAIS-4, BMS3, postop cognitive manifestations can be positive or negative or transient |  | 1 |  | 3.6 % |  | 64.3 % |  |
| We use a comprehensive battery for language, memory, attention, sensorimotor functions, executive functions and social cognition. The battery comprises standardised tests that are compiled on the basis of clinical experience and findings. Specific information is stored in the neuropsychology database module |  | 1 |  | 3.6 % |  | 67.9 % |  |
| Wechsler memory tests; especially in adults resecting the dominant hippocampus |  | 1 |  | 3.6 % |  | 71.4 % |  |
| as specified here: https://www.lice.it/pdf/Il_Trattamento_Chirurgico_Dell_Epilessia_080420.pdf |  | 1 |  | 3.6 % |  | 75.0 % |  |
| assessment of verbal and figural memory functions was performed using the German version of the Rey Auditory-Verbal Learning Test (Verbaler Lern- und Merkfähigkeitstest), a figural learning and memory test (Diagnosticum für Cerebralschädigung-II), and a face recognition test, Speech fluency and naming is assessed using the Regensburger Wortflüssigkeitstest and the Boston Naming Test. The examination normally includes tests for attention and speed performance (subtests of the Testbatterie zur Aufmerksamkeitsprüfung) and working memory tests (subtest of the revised Wechsler Memory Scale) |  | 1 |  | 3.6 % |  | 78.6 % |  |
| complete neuropsy testing |  | 1 |  | 3.6 % |  | 82.1 % |  |
| complex test battery |  | 1 |  | 3.6 % |  | 85.7 % |  |
| difficult to answer |  | 1 |  | 3.6 % |  | 89.3 % |  |
| psych department |  | 1 |  | 3.6 % |  | 92.9 % |  |
| standard neuropsych batteries performed by trained neuropsychologist |  | 1 |  | 3.6 % |  | 96.4 % |  |
| we systematically perform a complete neuropsychological testing before and several times after all types of epilepsy surgery in children, including the WISC5, Vineland scale, testing for verbal and visuo-spatial comprehension, fluidity of reasoning, working memory, processing speed |  | 1 |  | 3.6 % |  | 100.0 % |  |
|  | | | | | | | |

| Frequencies of 21. What is the observed frequency of speech and language disorders after _______? [ATL in children] | | | | | | | |
| --- | --- | --- | --- | --- | --- | --- | --- |
| **21. What is the observed frequency of speech and language disorders after _______?** | | **Counts** | | **% of Total** | | **Cumulative %** | |
| 0-2% |  | 30 |  | 76.9 % |  | 76.9 % |  |
| 3-5% |  | 6 |  | 15.4 % |  | 92.3 % |  |
| 6-10% |  | 3 |  | 7.7 % |  | 100.0 % |  |
|  | | | | | | | |

| Frequencies of 21. What is the observed frequency of speech and language disorders after _______? [selAH in children] | | | | | | | |
| --- | --- | --- | --- | --- | --- | --- | --- |
| **21. What is the observed frequency of speech and language disorders after _______?** | | **Counts** | | **% of Total** | | **Cumulative %** | |
| 0-2% |  | 22 |  | 88.0 % |  | 88.0 % |  |
| 3-5% |  | 3 |  | 12.0 % |  | 100.0 % |  |
|  | | | | | | | |

| Frequencies of 21. What is the observed frequency of speech and language disorders after _______? [ATL in adults] | | | | | | | |
| --- | --- | --- | --- | --- | --- | --- | --- |
| **21. What is the observed frequency of speech and language disorders after _______?** | | **Counts** | | **% of Total** | | **Cumulative %** | |
| 0-2% |  | 16 |  | 44.4 % |  | 44.4 % |  |
| 11-15% |  | 1 |  | 2.8 % |  | 47.2 % |  |
| 16-20% |  | 1 |  | 2.8 % |  | 50.0 % |  |
| 3-5% |  | 14 |  | 38.9 % |  | 88.9 % |  |
| 6-10% |  | 4 |  | 11.1 % |  | 100.0 % |  |
|  | | | | | | | |

| Frequencies of 21. What is the observed frequency of speech and language disorders after _______? [selAH in adults] | | | | | | | |
| --- | --- | --- | --- | --- | --- | --- | --- |
| **21. What is the observed frequency of speech and language disorders after _______?** | | **Counts** | | **% of Total** | | **Cumulative %** | |
| 0-2% |  | 16 |  | 59.3 % |  | 59.3 % |  |
| 11-15% |  | 1 |  | 3.7 % |  | 63.0 % |  |
| 3-5% |  | 9 |  | 33.3 % |  | 96.3 % |  |
| 6-10% |  | 1 |  | 3.7 % |  | 100.0 % |  |
|  | | | | | | | |

| Frequencies of 22. What is the minimally required surgical experience measured in number of cases for individually practicing [ATL in children] | | | | | | | |
| --- | --- | --- | --- | --- | --- | --- | --- |
| **22. What is the minimally required surgical experience measured in number of cases for individually practicing** | | **Counts** | | **% of Total** | | **Cumulative %** | |
| 10 cases |  | 7 |  | 17.5 % |  | 17.5 % |  |
| 20 cases |  | 19 |  | 47.5 % |  | 65.0 % |  |
| 30 cases |  | 10 |  | 25.0 % |  | 90.0 % |  |
| 40 cases |  | 2 |  | 5.0 % |  | 95.0 % |  |
| 50 cases or more |  | 2 |  | 5.0 % |  | 100.0 % |  |
|  | | | | | | | |

| Frequencies of 22. What is the minimally required surgical experience measured in number of cases for individually practicing [selAH in children] | | | | | | | |
| --- | --- | --- | --- | --- | --- | --- | --- |
| **22. What is the minimally required surgical experience measured in number of cases for individually practicing** | | **Counts** | | **% of Total** | | **Cumulative %** | |
| 10 cases |  | 5 |  | 17.2 % |  | 17.2 % |  |
| 20 cases |  | 11 |  | 37.9 % |  | 55.2 % |  |
| 30 cases |  | 8 |  | 27.6 % |  | 82.8 % |  |
| 40 cases |  | 3 |  | 10.3 % |  | 93.1 % |  |
| 50 cases or more |  | 2 |  | 6.9 % |  | 100.0 % |  |
|  | | | | | | | |

| Frequencies of 22. What is the minimally required surgical experience measured in number of cases for individually practicing [ATL in adults] | | | | | | | |
| --- | --- | --- | --- | --- | --- | --- | --- |
| **22. What is the minimally required surgical experience measured in number of cases for individually practicing** | | **Counts** | | **% of Total** | | **Cumulative %** | |
| 10 cases |  | 6 |  | 16.7 % |  | 16.7 % |  |
| 20 cases |  | 20 |  | 55.6 % |  | 72.2 % |  |
| 30 cases |  | 3 |  | 8.3 % |  | 80.6 % |  |
| 40 cases |  | 2 |  | 5.6 % |  | 86.1 % |  |
| 50 cases or more |  | 5 |  | 13.9 % |  | 100.0 % |  |
|  | | | | | | | |

| Frequencies of 22. What is the minimally required surgical experience measured in number of cases for individually practicing [selAH in adults] | | | | | | | |
| --- | --- | --- | --- | --- | --- | --- | --- |
| **22. What is the minimally required surgical experience measured in number of cases for individually practicing** | | **Counts** | | **% of Total** | | **Cumulative %** | |
| 10 cases |  | 4 |  | 13.8 % |  | 13.8 % |  |
| 20 cases |  | 11 |  | 37.9 % |  | 51.7 % |  |
| 30 cases |  | 9 |  | 31.0 % |  | 82.8 % |  |
| 40 cases |  | 4 |  | 13.8 % |  | 96.6 % |  |
| 50 cases or more |  | 1 |  | 3.4 % |  | 100.0 % |  |
|  | | | | | | | |
