# Supplementary material for: Anterior temporal lobectomy and selective AmygdaloHippocampectomy complications across Europe: review, meta-analysis, and Delphi consensus
Source: Brain Spine. 2025 Jun 18;5:104304. doi: 10.1016/j.bas.2025.104304 (PMC12272908; doi:10.1016/j.bas.2025.104304)
Supplement: Multimedia component 2 [file mmc2.docx]

**Questionnaire:**

**Anterior Temporal Lobectomy and selective AmygdaloHippocampectomy**

**complications across Europe: review, meta-analysis, and Deplhi consensus**

1. What is the observed mortality after _______?

|  | 0-1% | 2-10% | 11-15% | 16-20% | more than 20% |
| --- | --- | --- | --- | --- | --- |
| ATL in children |  |  |  |  |  |
| selAH in children |  |  |  |  |  |
| ATL in adults |  |  |  |  |  |
| selAH in adults |  |  |  |  |  |

2. What is the occurrence rate of visual deficits after _______?

|  | 0-2% | 3-5% | 6-10% | 11-15% | >16% |
| --- | --- | --- | --- | --- | --- |
| ATL in children |  |  |  |  |  |
| selAH in children |  |  |  |  |  |
| ATL in adults |  |  |  |  |  |
| selAH in adults |  |  |  |  |  |

3. Which of the following measures would you consider to avoid visual field deficits?

1. Pre-op DTI for identification of visual pathway
2. Intraoperative visual evoked potentials (VEPs)
3. Intra-operative stimulation for identification of visual pathway
4. Other

4. How would you detect visual deficits in your service routinely?

1. Clinical examination
2. Visual field examination
3. I don't routinely examine visual fields
4. Other

5. What is the observed frequency rate of permanent motor deficits rate after _______?

|  | 0-2% | 3-5% | 6-10% | 11-15% | 16-20% |
| --- | --- | --- | --- | --- | --- |
| ATL in children |  |  |  |  |  |
| selAH in children |  |  |  |  |  |
| ATL in adults |  |  |  |  |  |
| selAH in adults |  |  |  |  |  |

6. Which of the following measures would you routinely routinely to avoid motor deficits?

1. Pre-op fMRI
2. MEPs, ESSPs
3. Direct cortical and subcortical stimulation
4. Other

7. Would you routinely employ TCD to detect  postoperative vasospasm?

1. Yes
2. No
3. Other

8. What is the observed early (<30 days) infection rate after _______?

|  | 0-2% | 3-5% | 6-10% | 11-15% | 16-20% |
| --- | --- | --- | --- | --- | --- |
| ATL in children |  |  |  |  |  |
| selAH in children |  |  |  |  |  |
| ATL in adults |  |  |  |  |  |
| selAH in adults |  |  |  |  |  |

9. What is the late (>30 days) observed infection rate after _______?

|  | 0-2% | 3-5% | 6-10% | 11-15% | 16-20% |
| --- | --- | --- | --- | --- | --- |
| ATL in children |  |  |  |  |  |
| selAH in children |  |  |  |  |  |
| ATL in adults |  |  |  |  |  |
| selAH in adults |  |  |  |  |  |

10. Do you shave hairs before surgery?

1. Yes
2. No
3. Sometimes

11. Please describe your strategy on antibiotic prophylaxis for temporal lobectomy.

Your Answer:____________________________________ *(in this question the participants were given a certain answer box to fill in their responses)*

12. The duration of surgery has _________ on the incidence of postoperative infections. Choose the appropriate option to fill the gap.

1. a negative impact
2. a positive impact
3. no role
4. other

13. What is the observed occurrence rate of postoperative hematomas requiring surgical management?

|  | 0-5% | 6-10% | 11-15% | 16-20% | more than 20% |
| --- | --- | --- | --- | --- | --- |
| In children after ATL |  |  |  |  |  |
| In children after selAH |  |  |  |  |  |
| In adults after ATL |  |  |  |  |  |
| In adults after selAH |  |  |  |  |  |

14. Would you routinely use a drain to avoid postoperative hematomas?

1. Yes
2. No
3. Some times
4. Other

15. What is the frequency of psychiatric manifestations after _______?

|  | 0-2% | 3-5% | 6-10% | 11-15% | 16-20% |
| --- | --- | --- | --- | --- | --- |
| ATL in children |  |  |  |  |  |
| selAH in children |  |  |  |  |  |
| ATL in adults |  |  |  |  |  |
| selAH in adults |  |  |  |  |  |

16. Would you routinely employ pre- and postoperative psychiatric evaluation after temporal lobectomy?

1. Yes
2. No
3. Some times
4. Other

17. If you answered "Yes" in the previous question, please specify which tests or batteries would you employ?

Your answer:____________________________________ *(in this question the participants were given a certain answer box to fill in their responses)*

18. What is the observed frequency of cognitive manifestations after _______?

|  | 0-2% | 3-5% | 6-10% | 11-15% | 16-20% |
| --- | --- | --- | --- | --- | --- |
| ATL in children |  |  |  |  |  |
| selAH in children |  |  |  |  |  |
| ATL in adults |  |  |  |  |  |
| selAH in adults |  |  |  |  |  |

19. Would you routinely employ pre- and postoperative  neuropsychological examinations after lobectomy?

1. Yes
2. No
3. Some times
4. Other

20. If you answered yes in the previous question,  please specify which tests or batteries of tests would you employ?

21. What is the observed frequency of speech and language disorders after _______?

|  | 0-2% | 3-5% | 6-10% | 11-15% | 16-20% |
| --- | --- | --- | --- | --- | --- |
| ATL in children |  |  |  |  |  |
| selAH in children |  |  |  |  |  |
| ATL in adults |  |  |  |  |  |
| selAH in adults |  |  |  |  |  |

22. What is the minimally required surgical experience measured in number of cases for individually practicing

|  | 10 cases | 20 cases | 30 cases | 40 cases | 50 cases or more |
| --- | --- | --- | --- | --- | --- |
| ATL in children |  |  |  |  |  |
| selAH in children |  |  |  |  |  |
| ATL in adults |  |  |  |  |  |
| selAH in adults |  |  |  |  |  |
